# PLMA-b-POEGMA Amphiphilic Block Copolymers as Nanocarriers for the Encapsulation of Magnetic Nanoparticles and Indomethacin

**DOI:** 10.3390/polym10010014

**Published:** 2017-12-23

**Authors:** Athanasios Skandalis, Andreas Sergides, Aristides Bakandritsos, Stergios Pispas

**Affiliations:** 1 Theoretical and Physical Chemistry Institute, National Hellenic Research Foundation, 48 Vassileos Constantinou Avenue, 11635 Athens, Greece; thanos.skan@gmail.com; 2 Department of Pharmacy, University of Patras, 26504 Rio Patras, Greece; andreas.sergides.15@ucl.ac.uk; 3 Department of Materials Science, University of Patras, 25604 Rio Patras, Greece; 4 UCL Healthcare Biomagnetic and Nanomaterials Laboratories, Department of Physics and Astronomy, The Royal Institution of Great Britain, 21 Albemarle street, London W1S 4BS, UK; 5 Regional Centre of Advanced Technologies and Materials, Department of Physical Chemistry, Faculty of Science, Palacky University, Šlechtitelů 27, 783 71 Olomouc, Czech Republic; aristeidis.bakandritsos@upol.cz

**Keywords:** amphiphilic block copolymers, micelles, self-assembly, encapsulation, magnetic nanoparticles, indomethacin

## Abstract

We report here on the utilization of poly(lauryl methacrylate)-*b*-poly(oligo ethylene glycol methacrylate) (PLMA-*b*-POEGMA) amphiphilic block copolymers, which form compound micelles in aqueous solutions, as nanocarriers for the encapsulation of either magnetic iron oxide nanoparticles or iron oxide nanoparticles, and the model hydrophobic drug indomethacin in the their hydrophobic core. The mixed nanostructures were characterized using dynamic light scattering (DLS) and transmission electron microscopy (TEM) in terms of their structure and solution properties. Magnetophoresis experiments showed that the mixed solutions maintain the magnetic properties of the initial iron oxide nanoparticles. Results indicate that the cumulative hydrophilic/hydrophobic balance of all components determines the colloidal stability of the nanosystems. The effect of salt and bovine serum albumin (BSA) protein concentration on the structure of the mixed nanostructures was also investigated. Disintegration of the mixed nanostructures was observed in both cases, showing the importance of these parameters in the structure formation and stability of such complex mixed nanosystems.

## 1. Introduction

Polymer-based nanomedicine has gained vast attention in the worldwide scientific community because of the promising potential it provides in the fields of bioimaging and the therapy of various diseases and disorders. Theranostics is a new scientific term describing nanosystems used for applications in diagnosis and therapy at the same time [1,2]. The polymeric systems used for theranostics usually consist of the polymeric material, the pharmaceutical substance that is used for therapy purposes and a chemical group/component that is used for diagnostic purposes [3,4,5]. Amphiphilic block copolymers consist of two or more covalently connected macromolecular chains with different hydrophobicity. Therefore, these polymers are able to self-assemble into various complex nanostructures, such as spherical or cylindrical micelles and vesicles, where the hydrophobic polymer chains form the core and the hydrophilic polymer chains form the corona when inserted in aqueous solutions [6,7]. The morphology of the self-assemblies depends on the hydrophobic/hydrophilic ratio. This property renders them ideal materials for the encapsulation and delivery of hydrophobic drugs and imaging agents in the hydrophobic core [8,9,10,11,12,13,14]. The encapsulation of nanoparticles (NPs) in the self-assembled nanostructures of block copolymers can provide the resulting hybrid nanostructures with enhanced properties such as improved stability, easy multi-functionalization and reduced toxicity, combining the characteristics of both the nanoparticles and the polymers [15,16]. Nowadays, magnetic nanoparticles are key materials, as far as biomedical applications are concerned, in fields such as magnetic resonance imaging (MRI), magnetic hyperthermia and drug delivery systems for triggered release of the encapsulated drug [15,17,18,19,20,21]. Encapsulation of magnetic NPs in block copolymers micelles can lead to improved stability and biocombatibility [22].

The aim of this work was to utilize amphiphilic poly(lauryl methacrylate)-*b*-poly (oligo ethylene glycol methacrylate), (PLMA-*b*-POEGMA) micelles as nanocarriers for the encapsulation of iron oxide magnetic NPs (synthesized by thermal decomposition) and both NPs and indomethacin (IND, a model hydrophobic drug) simultaneously in their hydrophobic PLMA core. The structure and properties of the hybrid nanostructures are studied by a gamut of physicochemical techniques including dynamic light scattering (DLS) and transmission electron microscopy (TEM).

## 2. Materials and Methods

### 2.1. Materials

Tetrahydrofuran (THF, 99.9%, Sigma-Aldrich, Athens, Greece), *n*-Hexane (95%, Carlo-Erba, Athens, Greece), 1-octadecene (Alfa Aesar, Athens, Greece, 90%,), iron chloride hexahydrate (FeCl_3_·6H_2_O, Acros Organics, Athens, Greece, 99%,), sodium oleate (Sigma-Aldrich, Athens, Greece, 82%,), oleic acid (Alfa Aesar, Athens, Greece, 90%,), bovine serum albumin (BSA, 99%, Sigma-Alrdrich, Athens, Greece), sodium chloride (99.5%, Sigma-Arldrich, Athens, Greece), indomethacin (IND, 99%, Fluka, Athens, Greece).

### 2.2. PLMA-b-POEGMA Block Copolymers Synthesis

The PLMA-*b*-POEGMA block copolymers were synthesized in various compositions by a two-step Reversible Addition-Fragmentation Chain Transfer (RAFT) polymerization process using 2,2′-Azobis (Isobutyronitrile), (AIBN) as the radical initiator and 4-cyano-4-(phenyl-carbonothioylthio) pentanoic acid (CPAD) as the chain transfer agent. The synthetic procedure has been described in detail in our previous work [23].

### 2.3. Synthesis of Iron Oxide Nanoparticles

Iron oxide NPs were synthesized by thermal decomposition of iron-oleate complex (precursor) in the presence of oleic acid and 1-octadecene.

(a) Synthesis of the precursor: The synthesis of the precursor was performed according to a procedure reported in the literature [24]. In a typical synthesis, iron chloride hexahydrate (FeCl_3_·6H_2_O, 8.1 g, 30 mmol) and sodium oleate (27.4 g, 90 mmol) were dissolved in a mixture of 60 mL absolute ethanol and 45 mL distilled water, followed by the addition of 105 mL hexane. The mixture was then refluxed (70 °C) for 4 h. Thereafter, the upper organic layer containing the iron-oleate complex (red-brown) was washed three times with 50 mL distilled water in a separatory funnel. In order to remove any residual water, sodium sulphate anhydrous (0.5–1.0 g) was added to the solution of the precursor, followed by filtration. Hexane was evaporated off in a rotary evaporator and the resulting waxy-solid iron-oleate complex was further dried for 2 h.

(b) Synthesis of Fe_2_O_3_ NPs: (3.6 g, 4.0 mmol) iron-oleate complex and (3.4 g, 12 mmol) oleic acid were dissolved in 30 g, 38 mL 1-octadecene. The mixture was heated to 100 °C for 30 min and then to reflux (318 °C) for 1 h (heating rate: 7 °C/min). The resulting black dispersion containing the NPs was left to cool down to room temperature and washed four times with a mixture of chloroform and acetone (1:3–1:1). At every wash, the nanocrystals were first dispersed in chloroform (CHCl_3_) and then precipitated by addition of acetone and centrifugation. Finally, the NPs were dispersed in CHCl_3_. The procedure described above, led to the formation of monodisperse nanocrystals with an average diameter of 9.4 nm determined by TEM (JEOL, Akishima, Tokyo, Japan).

### 2.4. Preparation of PLMA-b-POEGMA Micelles Loaded with Magnetic Nanoparticles

The amphiphilic block copolymer PLMA-*b*-POEGMA was dissolved in THF at a concentration of 5 × 10^−3^ g/mL. A dispersion of iron oxide nanoparticles in CHCl_3_, stabilized with oleic acid, was added to the solution. The concentration of magnetic nanoparticles in the mixture was set to 10 or 20 wt % relative to the PLMA block. Then the mixture was injected fast in 10 mL distilled water under vigorous stirring. Thereafter, the mixed solution was placed in a rotary evaporator in order to evaporate the organic solvents (THF and CHCl_3_). The mixed micelles with encapsulated NPs were generated during the evaporation of the organic solvents. The loading percentage in all cases was more than 95%, since no precipitate was observed, at least for the stable solutions.

### 2.5. Preparation of PLMA-b-POEGMA Micelles Loaded with Magnetic Nanoparticles and Indomethacin

The amphiphilic block copolymer PLMA-*b*-POEGMA was dissolved in THF at a concentration of 5 × 10^−3^ (g/mL). The appropriate amount of indomethacin was dissolved in THF. A dispersion of iron oxide nanoparticles in CHCl_3_, stabilized with oleic acid and indomethacin in THF, were added to the solution. The concentration of magnetic nanoparticles in the mixture was 10 wt % and the concentration of indomethacin was 20 wt % relative to the PLMA block. Then, the mixture was injected quickly in 10 mL distilled water under vigorous stirring. Thereafter, the mixed solution was placed in a rotary evaporator in order to evaporate the organic solvents. The mixed micelles with encapsulated NPs and IND were generated during the evaporation of the organic solvents. The loading percentage in all cases was more than 95%, as evidenced by the absence of precipitates and UV–Vis (Perkin Elmer, Waltham, MA, USA) characterization.

### 2.6. Methods

Dynamic light scattering measurements were conducted on an ALV/CGS-3 compact goniometer system (ALVGmbH, Hessen, Germany), equipped with an ALV 5000/EPP multi-τ digital correlator with 288 channels and an ALV/LSE-5003 light scattering electronics unit for stepper motor drive and limit switch control. A JDS Uniphase 22 mW He-Ne laser (λ = 632.8 nm) was used as the light source. Measurements were carried out five times for each concentration and angle and were averaged for each angle. The solutions were filtered through 0.45 μm hydrophilic PTFE filters (Millex-LCR from Millipore, Billerica, MA, USA) before measurements. The angular range for the measurements was 30–150°. Obtained correlation functions were analyzed by the cumulants method and CONTIN software (ALVGmbH, Hessen, Germany). The size data and figures shown below are from measurements at 90°.

The magnetophoretic experiments were performed using a Perkin Elmer (Lambda 19) UV–Vis–NIR spectrophotometer (Waltham, MA, USA) and by inserting next to the cuvette holder a cylindrical Nd-Fe-B magnet (dimensions: diameter = 20 mm, thickness = 10 mm, magnetization unit: N45, attraction/repulsion strength: max 16 kg). The wavelength of the measurements was set at 450 nm and the absorbance of the solution containing the hybrid nanostructures with encapsulated NPs was measured for 1 h under the influence of the magnet. It should be noted that absorption in this wavelength comes from the encapsulated NPs and not from the copolymers.

TEM micrographs were obtained by a JEOL, JEM-2100 instrument (Akishima, Tokyo, Japan) operating at 200 kV. Samples for TEM were prepared by casting a droplet of a dilute aqueous suspension (ca. 1 × 10^−4^ g·mL^−1^) of the mixed micelles on copper grids coated by Formvar carbon film.

## 3. Results and Discussion

The self-assembly of PLMA-*b*-POEGMA amphiphilic block copolymers has been studied extensively in our previous work [23]. In aqueous solutions, the block copolymers have been found to form compound micelles where the PLMA block is forming the hydrophobic domains and POEGMA the hydrophilic corona.

**Scheme 1:**
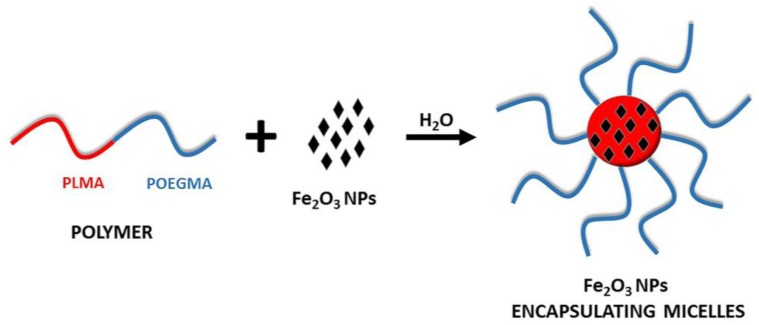
Simplified schematic illustration depicting the preparation of the PLMA-*b*-POEGMA/NPs mixed nanostructures.

In order to impart magnetic properties to the polymeric micelles, small size (*D* = 9.4 nm) iron oxide nanoparticles stabilized with oleic acid were encapsulated in the hydrophobic core of the micelles. It was expected that the oleic acid corona of NPs is compatible and miscible with the lauryl side chains of the PLMA block and, in this way, the encapsulation of the magnetic NPs would be greatly facilitated. Moreover, both indomethacin and NPs could be simultaneously encapsulated in the micellar core in order to create nanocarrier systems suitable for both imaging and therapy. Thus, PLMA is the interacting functional block that promotes the miscibility of the components. On the other hand, POEGMA blocks provide solubility/colloidal stability and stealth properties to the mixed nanostructures. Table 1 shows the molecular characteristics of the PLMA-*b*-POEGMA block copolymers, as well as the DLS results for all mixed nanostructures. In all cases, mixed structures within the nanoscale range, with a relatively narrow size distribution were obtained. Scheme 1 depicts the procedure followed for the preparation of the micelles loaded only with magnetic NPs.

### 3.1. Encapsulation of Fe_2_O_3_ Nanoparticles in the PLMA-b-POEGMA Micelles

**Figure 1:**
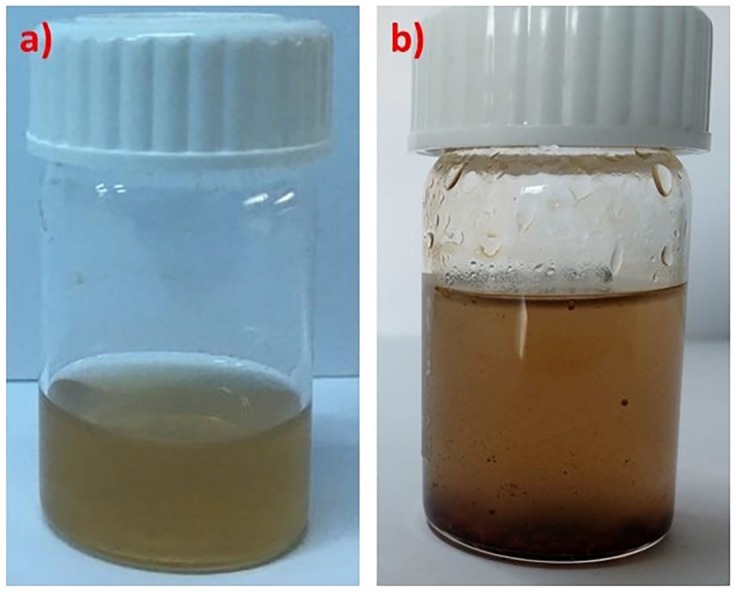
Colloidally stable (**a**) and unstable (**b**) mixed solutions of PLMA_22_-*b*-POEGMA_32_ micelles containing 10 and 20 wt % of magnetic nanoparticles (NPs) relative to PLMA, respectively.

A major concern when it comes to the preparation of such mixed nanosystems is the colloidal stability of the mixed solutions. This was possible only for PLMA_22_-*b*-POEGMA_32_ and PLMA_22_-*b*-POEGMA_13_ containing 10 wt % NPs with regard to PLMA mass. In the case of PLMA_22_-*b*-POEGMA_58_, the magnetic NPs precipitated, probably due to the low PLMA hydrophobic content relative to POEGMA, which apparently is not enough to accommodate a significant amount of the NPs. Trials for the encapsulation of greater amounts of NPs were carried out, but the mixed solutions were not colloidally stable, with only a small fraction of the NPs being encapsulated in the form of an aqueous suspension, while most of the materials existed as precipitates at the bottom of the vials (Figure 1).

**Figure 2:**
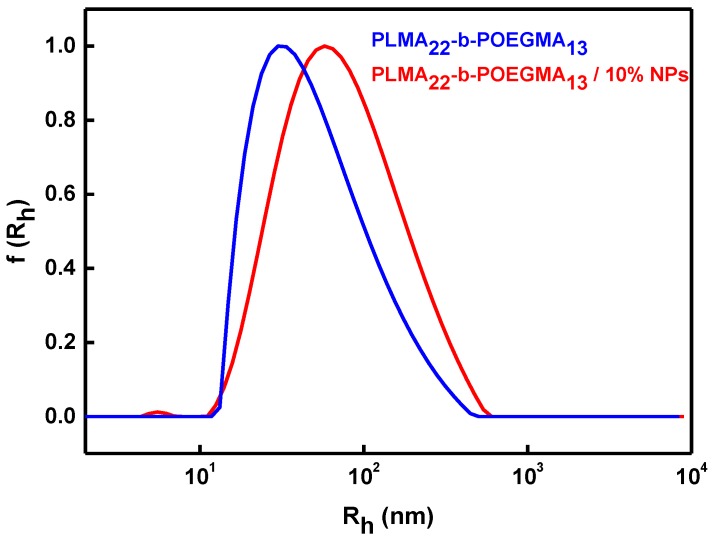
Comparative size distribution graphs from CONTIN analysis before and after the encapsulation of the magnetic NPs within the PLMA_22_-*b*-POEGMA_13_ micelles.

In order to investigate the size of the NPs encapsulating micelles, dynamic light scattering was utilized. In Figure 2, a typical size distribution graph from CONTIN analysis before and after the encapsulation of the magnetic NPs is shown. The results indicate that the Fe_2_O_3_ NPs affect the size of the mixed nanostructures, and the hydrodynamic diameter (*D*_h_) is significantly larger after the encapsulation of the magnetic NPs (Table 1). This is a result of the fact that a larger amount of hydrophobic components exists in the mixed nanostructures, and a larger amount of block copolymer participates in the organization of the materials in order to provide a colloidally stable nanosystem.

**Figure 3:**
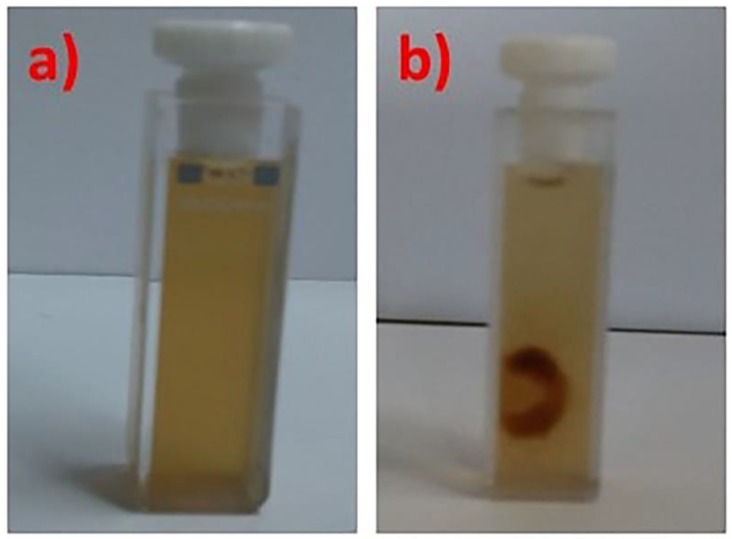
PLMA_22_-*b*-POEGMA_13_/10% NPs mixed solution before (**a**) and after (**b**) the application of magnetic field (using a magnet) for the magnetophoresis experiments.

Magnetophoretic experiments were performed in order to investigate whether the iron oxide NPs containing mixed nanostructures maintain the magnetic properties of the inorganic part after the encapsulation in the polymeric micelles. As can be seen in Figure 3, the magnetic mixed colloidal nanostructures are gathered in the side of the measuring cell in the place where the magnetic field was applied.

**Figure 4:**
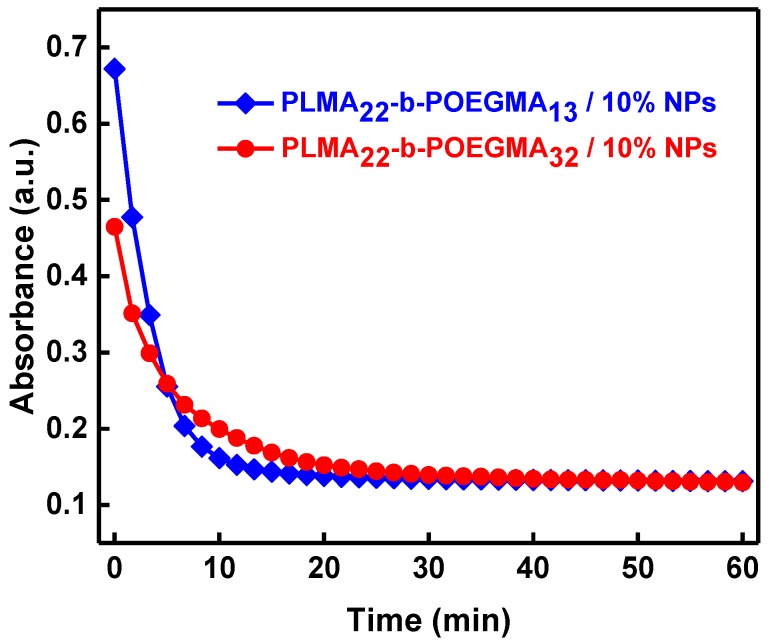
Magnetophoresis graphs measuring absorbance as a function of time (λ = 450 nm) for PLMA_22_-*b*-POEGMA_32_ and PLMA_22_-*b*-POEGMA_58_ micelles loaded with 10 wt % magnetic NPs in regard to PLMA mass in the mixed nanosystem.

In addition, in the magnetophoretic experiments performed with the aid of a UV–Vis spectrophotometer, there is a decrease in the absorbance at 450 nm because the magnetic material gathers in the side where the magnet is placed. The magnetophoresis graphs shown in Figure 4 prove that the NPs loaded micelles have a strong response to the application of the external magnetic field, since the absorbance of the solution decreases rapidly within the first ten minutes of the measurement and then shows a small and more gradual decrease up to approximately 20 min. No further decrease in the absorbance can be observed in the remaining time of the experiment. It seems that the phenomenon is faster for the PLMA_22_-*b*-POEGMA_13_/10% NPs mixed nanosystem, which obviously contains the higher amount of the magnetic material (as can be deduced by the stoichiometry of the solutions and from the initial value of the absorbance).

**Figure 5:**
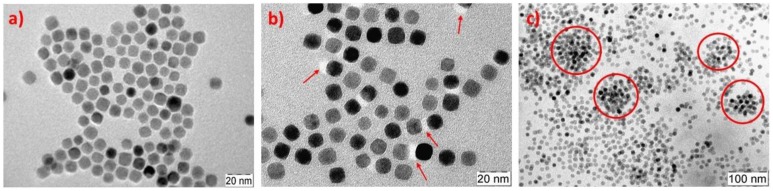
TEM images for (**a**) iron oxide NPs; (**b**) PLMA_22_-*b*-POEGMA_32_/10% NPs (red arrows indicate areas with a higher concentration of polymeric chains) and (**c**) PLMA_22_-*b*-POEGMA_13_/10% NPs mixed nanosystems after solvent evaporation (red circles indicate areas with large aggregation).

In order to have a more complete picture of the morphology of the iron oxide NPs before and after their loading in the polymeric micelles, TEM was utilized as a nanoscale imaging technique. At this point, it should be noted that the contrast in the mixed systems studied is mainly due to the inorganic NPs, and the polymeric material is barely visible in the micrographs. In Figure 5a, it can be observed that the iron oxide NPs in the absence of copolymer are in some way oriented to the carbon coated grid after the evaporation of the solvent, and this ordering is determined by the oleic acid ligands on the NPs surface. The mean distance between the particles is approximately 1.9 nm, which corresponds rather well with double the space of an oleic acid tail. In Figure 5b, where the block copolymer PLMA_22_-*b*-POEGMA_32_ is involved, it can be observed that the mean distance between the particles is significantly larger (ca. 4.6 nm); a fact that leads to the conclusion that the surface of the NPs is decorated with the block copolymer chains. This idea is enhanced from the observation of white shadows in the TEM images (noted with red arrows) which must be areas with a higher concentration of polymeric chains (or parts of polymeric spherical micelles). In the case of a PLMA_22_-*b*-POEGMA_13_-based mixed nanosystem, similar areas can be observed, but also areas with large aggregation of magnetic NPs (red circles); an observation that indicates both the encapsulation of the magnetic NPs within polymeric micelles and the decoration of NPs surface with block copolymer chains. Most probably, the length of the PLMA block and its content dictate the interactions between block copolymer chains and the NPs, as well as the formation of mixed nanostructures in aqueous media. On the other hand, a restructuring of the mixed nanostructures on the carbon surface of the TEM grid in the dry state cannot be ruled out due to the low *T*_g_ of the PLMA block. Therefore, there is a possibility that the structures observed by DLS and TEM are not exactly the same, due to the different state of the material during the measurements (suspension vs. dry state). In any case, TEM micrographs also show the presence of interactions between PLMA-*b*-POEGMA copolymers and magnetic NPs and their nanoscale organization. However, the general picture for the nanosystems obtained from DLS measurements is closer to the environments found in biomedical applications such as drug delivery, since they are mostly related to solutions or suspensions.

### 3.2. Encapsulation of Both Iron Oxide Nps and IND into PLMA-b-POEGMA Micelles

**Scheme 2:**
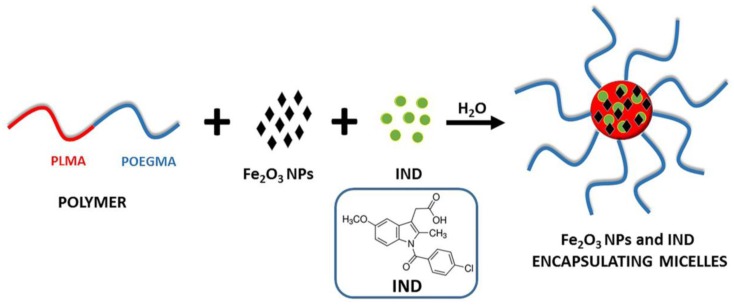
Simplified schematic illustration for the preparation of the PLMA-*b*-POEGMA/NPs/IND mixed nanostructures.

The next step was to investigate whether the polymeric micelles could encapsulate a hydrophobic drug together with the magnetic NPs in their PLMA core forming a three component colloidal nanosystem. The procedure followed for the preparation of PLMA-*b*-POEGMA micelles loaded with iron oxide NPs and the non-steroidal, anti-inflammatory drug indomethacin is depicted in Scheme 2.

This was achieved only for sample PLMA_22_-*b*-POEGMA_58_ in terms of colloidal stability. As stated above, this block copolymer was not able to form stable solutions with encapsulated iron oxide NPs alone. Probably, the polar groups contained in the hydrophobic IND molecule make it act as a low molecular surfactant, affecting the structural characteristics and colloidal stability of the mixed self-assembled nanostructures formed [23,25,26,27]. Regarding the rest of the mixed systems, PLMA_22_-*b*-POEGMA_32_/NPs/IND was stable for 72 h and PLMA_22_-*b*-POEGMA_13_/NPs/IND precipitated almost immediately after mixing the initial solutions. This fact indicates that the hydrophilic/hydrophobic balance, taking into account all components utilized in the preparation of the mixed nanostructures, is responsible for the stability of the final solution.

**Figure 6:**
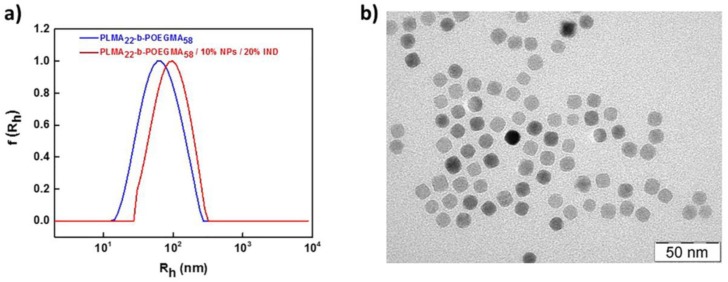
(**a**) Comparative size distribution graphs from CONTIN analysis before and after the encapsulation of magnetic NPs and IND in PLMA_22_-*b*-POEGMA_58_ micelles; (**b**) TEM micrograph for PLMA_22_-*b*-POEGMA_58_/10% NPs/20% IND.

DLS was utilized for the determination of the size distribution of the mixed PLMA-*b*-POEGMA/NPs/IND nanostructures. For the PLMA_22_-*b*-POEGMA_58_ system, the size is significantly larger than before the encapsulation (Figure 6a), whereas for PLMA_22_-*b*-POEGMA_32_ it is almost the same with the system where only magnetic NPs were encapsulated in the copolymer. This may be another proof for the surfactant-like activity of indomethacin in the cases under investigation. TEM observations (Figure 6b) show the dominating contrast of magnetic NPs and no substantial changes in the morphology compared with the systems where IND is absent (Figure 5b,c).

**Figure 7:**
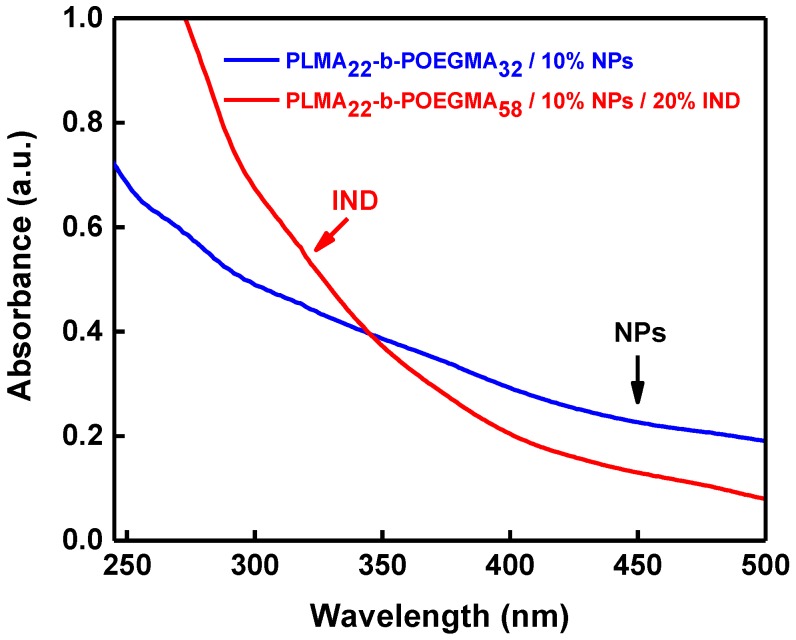
UV–Vis spectra of PLMA_22_-POEGMA_32_/10% NPs (blue line) and PLMA_22_-*b*-POEGMA_58_/10% NPs/20% IND (red line).

Further proof for the incorporation of IND in the polymeric micelles is given by the UV–Vis measurements shown in Figure 7. The increase in absorbance at 450 nm is attributed to the existence of the magnetic NPs in the micellar core, and the one at 321 nm is attributed to the presence of encapsulated indomethacin^27^.

**Figure 8:**
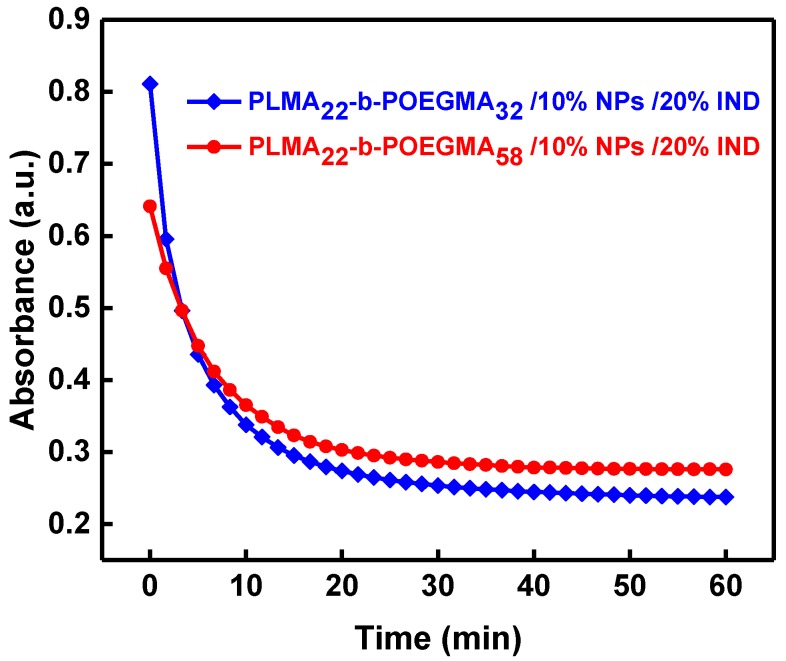
Magnetophoresis graphs showing absorbance as a function of time (λ = 450 nm) for PLMA_22_-*b*-POEGMA_58_ and PLMA_22_-*b*-POEGMA_32_ loaded with 10% magnetic NPs and 20% IND in respect to PLMA mass.

Magnetophoretic measurements were also performed in order to investigate the behavior of the three-component mixed nanostructures under the application of a magnetic field (Figure 8). The obtained results are similar to those for nanosystems that have only NPs encapsulated. This shows that the presence of IND does not influence the magnetic properties of the mixed nanostructures.

**Figure 9:**
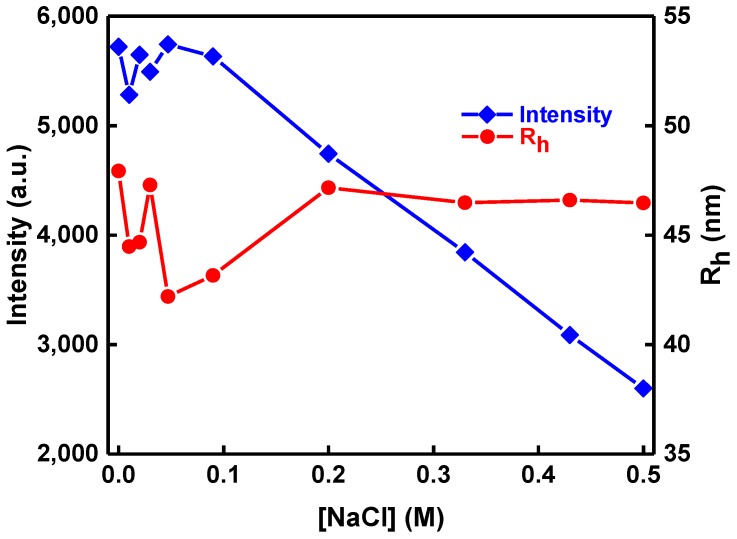
*R*_h_ and scattered intensity as a function of ionic strength ([NaCl]) for PLMA_22_-*b*-POEGMA_58_ /10% NPs/20% IND mixed aqueous solution.

Solution ionic strength is another parameter that affects the properties of aqueous solutions of nanoparticles. The effect of ionic strength in the structure of the PLMA-*b*-POEGMA/NPs/IND mixed nanosystems in aqueous solutions was also investigated by gradually increasing the concentration of NaCl (by addition of a 1 M NaCl stock solution) and subsequent DLS measurements on the resulting solutions. As can be observed in Figure 9, scattering intensity initially remains constant for ionic strengths up to ca. 0.1 M NaCl and then has the tendency to decrease as the concentration of salt increases. This observation supports a decrease in the mass of the mixed nanostructures by increasing ionic strength above 0.1 M NaCl. However, the determined hydrodynamic radius remains practically the same in the whole range investigated, revealing an absence of aggregation phenomena and pointing towards disintegration of the nanostructures accompanied by swelling (increase in the volume of the nanostructures). This may be related to phenomena taking place at the surface of the magnetic NPs, where an increase of ionic strength may result in a decrease of the oleic acid/Fe_2_O_3_ interactions and a decrease of the bound amount of stabilizing ligands, which in turn affects copolymer/NPs interactions, since the gluing components/interactions are eventually diminished.

**Figure 10:**
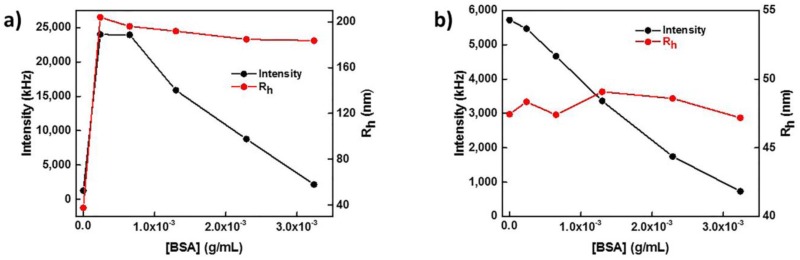
*R*_h_ and scattered intensity as a function of BSA concentration for PLMA_22_-*b*-POEGMA_58_ (**a**) and PLMA_22_-*b*-POEGMA_58_/10% NPs/20% IND (**b**) mixed nanostructures in water.

Having in mind the potential application of the mixed nanostructures in biological environments and most probably that of blood serum, the interaction of BSA with the mixed nanoassemblies was investigated by titration with aqueous BSA solution (1 × 10^−3^ g/mL stock solution concentration) and subsequent DLS measurements. The results are presented in Figure 10b. The scattered intensity decreases as the concentration of BSA increases, and there are no significant changes in the *R*_h_ values. The behavior depicted in Figure 10b indicates a decrease in the mass of the nanostructures accompanied by swelling of the structures, as was the case for the variation of the solution ionic strength.

In Figure 10a, the behavior of neat PLMA_22_-*b*-POEGMA_58_ micelles is depicted in the presence of BSA. It can be seen that both scattering intensity and size of the pure polymer micelles solutions increase rapidly after the first addition of BSA in the micellar solution. This means that BSA is adsorbed/incorporated in the polymeric micelles, resulting to the formation of higher aggregates. As the concentration of BSA increases, the values of *R*_h_ remain relatively constant but there is a decrease in the values of scattered intensity. This decrease in the intensity can be related to a decrease in the mass of the PLMA_22_-*b*-POEGMA_58_ micelles along with nanostructure swelling and that may explain the non-significant changes in the size after the initial addition of BSA. The phenomenon of the rapid increase in the size and intensity is not observed after the encapsulation of NPs and IND in the polymeric micelles, probably because, in the latter case, the structure of PLMA_22_-*b*-POEGMA_58_/10% NPs/20% IND assemblies is more compact and more stable, due to increased mainly hydrophobic interactions of the components, compared to that of neat PLMA_22_-*b*-POEGMA_58_ compound micelles. Apparently, BSA leads to partial disintegration of the mixed nanostructures, through incorporation of the protein within the newly formed PLMA_22_-*b*-POEGMA_58_/NPs/IND/BSA assemblies, due to the amphiphilic character of BSA, and its ability to interact with and to encapsulate hydrophobic compounds in the blood environment [28].

## 4. Conclusions

To summarize, the ability of poly(lauryl methacrylate)-*b*-poly(oligo ethylene glycol methacrylate) (PLMA-*b*-POEGMA) amphiphilic block copolymer self-assemblies in aqueous solutions to encapsulate magnetic iron oxide nanoparticles was investigated. These block copolymers self-assemble in compound micelles in aqueous solutions and can load up to 10 wt % iron oxide nanoparticles (NPs) in their PLMA cores forming mixed nanostructures. The size distribution of the mixed micelles is significantly broader after the loading of the magnetic NPs, as dynamic light scattering (DLS) results have shown. The NPs maintain their magnetic properties after the encapsulation in the micelles as it was proved by magnetophoretic measurements. The mixed solutions are colloidally stable for copolymers where the hydrophobic ratio is larger than 30 wt %.

In the case where both indomethacin (IND) and magnetic NPs were simultaneously loaded in the micellar core, only the lowest hydrophobic ratio copolymer was able to assure colloidal stability of the mixed three-component aggregates. The magnetic properties of the iron oxide NPs were also maintained after the encapsulation of the drug, resulting in magnetically active mixed nanostructures with encapsulated IND. The mixed aggregates seem to be affected by the presence of increased concentrations of salt and BSA but without loss of their nanosized dimensions and colloidal stability.

The colloidal stability, drug encapsulation ability and magnetic properties of the nanosystems based on biocompatible PLMA-*b*-POEGMA amphiphilic block copolymers prepared in this study hold potential for utilization of these hybrid nanostructures as drug delivery and triggered release systems, as well as for bioimaging applications.

## Figures and Tables

**Table 1:** Molecular characteristics and dynamic light scattering (DLS) results for poly(lauryl methacrylate)-*b*-poly(oligo ethylene glycol methacrylate) (PLMA-*b*-POEGMA) block copolymers and solutions of their micelles loaded with nanoparticles (NPs) and both indomethacin (IND) and NPs.

Block copolymers						NPs loaded		NPs and IND loaded	
Sample	*M*_w_^a^ (×10^4^) (g/mol)	*M*_w_/*M*_n_^a^	wt % PLMA ^b^	*D*_h_^c^ (nm)	PDI ^c^	*D*_h_^c^ (nm)	PDI ^c^	*D*_h_^c^ (nm)	PDI ^c^
PLMA_22_-*b*-POEGMA_58_	3.3	1.30	16	162	0.28	-	-	166	0.33
PLMA_22_-*b*-POEGMA_32_	2.07	1.31	27	122	0.35	198	0.37	194	0.39
PLMA_22_-*b*-POEGMA_13_	1.17	1.18	52	140	0.31	200	0.35	-	-

^a^ Determined by size exclusion chromatography (SEC), ^b^ Determined by nuclear magnetic resonance (^1^H NMR), ^c^ Determined by dynamic light scattering (DLS) at 90°.

## References

[1] Lammers T., Kiessling F., Hennink W.E., Storm G. (2010). Nanotheranostics and Image-Guided Drug Delivery: Current Concepts and Future Directions. Mol. Pharm..

[2] Kelkar S.S., Reineke T.M. (2011). Theranostics: Combining Imaging and Therapy. Bioconj. Chem..

[3] Krasia-Christoforou T., Georgiou T.K. (2013). Polymeric theranostics: Using polymer-based systems for simultaneous imaging and therapy. J. Mater. Chem. B.

[4] Talelli M., Rijcken C.J.F., Lammers T., Seevinck P.R., Storm G., van Nostrum C.F., Hennink W.E. (2009). Superparamagnetic Iron Oxide Nanoparticles Encapsulated in Biodegradable Thermosensitive Polymeric Micelles: Toward a Targeted Nanomedicine Suitable for Image-Guided Drug Delivery. Langmuir.

[5] Zahraei M., Marciello M., Lazaro-Carrillo A., Villanueva A., Herranz F., Talelli M., Costo R., Monshi A., Shahbazi-Gahrouei D., Amirnasr M. (2016). Versatile theranostics agents designed by coating ferrite nanoparticles with biocompatible polymers. Nanotechnology.

[6] Mai Y., Eisenberg A. (2012). Self-assembly of block copolymers. Chem. Soc. Rev..

[7] Tritschler U., Pearce S., Gwyther J., Whittell G.R., Manners I. (2017). 50th Anniversary Perspective: Functional Nanoparticles from the Solution Self-Assembly of Block Copolymers. Macromolecules.

[8] Fairbanks B.D., Gunatillake P.A., Meagher L. (2015). Biomedical applications of polymers derived by reversible addition—Fragmentation chain-transfer (RAFT). Adv. Drug Deliv. Rev..

[9] Duncan R. (2003). The dawning era of polymer therapeutics. Nat. Rev. Drug Discov..

[10] Aliabadi H.M., Lavasanifar A. (2006). Polymeric micelles for drug delivery. Expert Opin. Drug Deliv..

[11] Gaucher G., Dufresne M.-H., Sant V.P., Kang N., Maysinger D., Leroux J.-C. (2005). Block copolymer micelles: Preparation, characterization and application in drug delivery. J. Control. Release.

[12] Miyata K., Christie R.J., Kataoka K. (2011). Polymeric micelles for nano-scale drug delivery. React. Funct. Polym..

[13] Jones M.-C., Leroux J.-C. (1999). Polymeric micelles—A new generation of colloidal drug carriers. Eur. J. Pharm. Biopharm..

[14] Pinto Reis C., Neufeld R.J., Ribeiro J.A., Veiga F. (2006). Nanoencapsulation I. Methods for preparation of drug-loaded polymeric nanoparticles. Nanomedicine NBM.

[15] Wang J., Li W., Zhu J. (2014). Encapsulation of inorganic nanoparticles into block copolymer micellar aggregates: Strategies and precise localization of nanoparticles. Polymer.

[16] Yuan J., Müller A.H.E. (2010). One-dimensional organic–inorganic hybrid nanomaterials. Polymer.

[17] Kim J., Lee J.E., Lee S.H., Yu J., Lee J.H., Park T.G., Hyeon T. (2008). Designed Fabrication of a Multifunctional Polymer Nanomedical Platform for Simultaneous Cancer—Targeted Imaging and Magnetically Guided Drug Delivery. Adv. Mater..

[18] Zhang L., Gu F., Chan J., Wang A., Langer R.S., Farokhzad O.C. (2008). Nanoparticles in Medicine: Therapeutic Applications and Developments. Clin. Pharmacol. Ther..

[19] Laurent S., Forge D., Port M., Roch A., Robic C., Vander Elst L., Muller R.N. (2008). Magnetic Iron Oxide Nanoparticles: Synthesis, Stabilization, Vectorization, Physicochemical Characterizations, and Biological Applications. Chem. Rev..

[20] Kim J., Piao Y., Hyeon T. (2009). Multifunctional nanostructured materials for multimodal imaging, and simultaneous imaging and therapy. Chem. Soc. Rev..

[21] Hua X., Yang Q., Dong Z., Zhang J., Zhang W., Wang Q., Tan S., Smyth H.D.C. (2017). Magnetically triggered drug release from nanoparticles and its applications in anti-tumor treatment. Drug Deliv..

[22] Mahmoudi M., Hosseinkhani H., Hosseinkhani M., Boutry S., Simchi A., Journeay W.S., Subramani K., Laurent S. (2011). Magnetic Resonance Imaging Tracking of Stem Cells in Vivo Using Iron Oxide Nanoparticles as a Tool for the Advancement of Clinical Regenerative Medicine. Chem. Rev..

[23] Skandalis A., Pispas S. (2017). PLMA-b-POEGMA amphiphilic block copolymers: Synthesis and self-assembly in aqueous media. J. Polym. Sci. A.

[24] Park J., An K., Hwang Y., Park J.G., Noh H.J., Kim J.Y., Park J.H., Hwang N.M., Hyeon T. (2004). Ultra-large-scale syntheses of monodisperse nanocrystals. Nat. Mater..

[25] Pispas S., Hadjichristidis N. (2003). Aggregation Behavior of Poly(butadiene-b-ethylene oxide) Block Copolymers in Dilute Aqueous Solutions:  Effect of Concentration, Temperature, Ionic Strength, and Type of Surfactant. Langmuir.

[26] Raffa P., Wever D.A.Z., Picchioni F., Broekhuis A.A. (2015). Polymeric Surfactants: Synthesis, Properties, and Links to Applications. Chem. Rev..

[27] Nagy M., Szöllösi L., Kéki S., Faust R., Zsuga M. (2009). Poly(vinyl alcohol)-based Amphiphilic Copolymer Aggregates as Drug Carrying Nanoparticles. J. Macromol. Sci. A.

[28] Zhao Y., Marcel Y.L. (1996). Serum Albumin Is a Significant Intermediate in Cholesterol Transfer between Cells and Lipoproteins. Biochemistry.

